# A widespread single amino acid mutation in AcrA reduces tigecycline susceptibility in *Klebsiella pneumoniae*

**DOI:** 10.1128/spectrum.02030-23

**Published:** 2023-11-30

**Authors:** Yingchao Xu, Wenjia Wang, Wenya Su, Mingyu Wang, Hai Xu, Xuhua Zhang, Ling Li

**Affiliations:** 1 State Key Laboratory of Microbial Technology, Microbial Technology Institute, Shandong University, Qingdao, China; 2 Laboratory Medicine Center, The Second Hospital of Shandong University, Jinan, China; Beijing Institute of Genomics, Chinese Academy of Sciences, Beijing, China

**Keywords:** tigecycline non-susceptibility, *Klebsiella pneumoniae*, AcrA mutation, resistance-nodulation-division (RND) pump, sequence types

## Abstract

Whole-genome sequencing of a clinical tigecycline non-susceptible *Klebsiella pneumoniae* strain reveals a novel T188A mutation in AcrA, raising awareness on its role in tigecycline resistance. Through structural analysis of AcrAB-TolC in *Escherichia coli* that is highly homologous to *K. pneumoniae* AcrAB-TolC*,* weaker hydrogen-bond interaction of the AcrA_A188_ with lipoyl domain than AcrA_T188_ was found, which may provide greater flexibility for substrate translocation. In order to explore the resistance mechanism of this mutation, wildtype and T188A mutant of *acrA* were cloned in pACYC184 and transformed into *acrA* deleted *K. pneumoniae*. Several experiments, including measurement of MICs for tigecycline, survival assays, and determination of intracellular tigecycline content, were performed and suggested that T188A mutation in AcrA led to increased tigecycline MIC, improved strain survival under tigecycline stress, and decreased intracellular tigecycline concentrations. Further examination of prevalence of this mutation showed its surprisingly wide presence, particularly in hypervirulent multidrug-resistant *K. pneumoniae* sequence types. These results support the role of T188A as a widespread AcrA mutation reducing tigecycline susceptibility in *K. pneumoniae* through increasing tigecycline efflux and improving tigecycline tolerance.

**IMPORTANCE:**

Tigecycline, a glycecycline antibiotic with broad-spectrum activity against almost all Gram-positive and Gram-negative bacteria, is a highly concerned “last-resort” antibiotic. In addition to plasmid-hosted mobile *tet*(X) conferring high-level resistance to tigecycline, there are many reports suggesting increased expression of AcrAB-TolC efflux pump leads to tigecycline non-susceptibility. However, the role of mutations in AcrAB-TolC on tigecycline resistance has not been identified. This study reports a novel T188A mutation of the AcrA subunit of AcrAB-TolC complex in a clinical tigecycline-resistant *Klebsiella pneumoniae* strain and reveals the role of AcrA mutation on tigecycline resistance in *K. pneumoniae*. High prevalence of A188 type AcrA in hypervirulent multidrug-resistant *K. pneumoniae* indicates that mutations of the AcrAB-TolC complex may play a larger role in determining bacterial pathogenesis and antibiotic susceptibility than previously expected.

## INTRODUCTION

The rapid increase of bacterial resistance has become one of the urgent problems in the field of medicine and health in the 21st century. The effective treatment of serious bacterial infections caused by multidrug-resistant (MDR) bacteria, especially by extensively drug-resistant (XDR) and pandrug-resistant strains, relies on the application of “last-resort” antibiotics. Unfortunately, the presence of resistant genes, such as *bla*_NDM_ and *mcr*-1, greatly decreased the efficacy of carbapenems and colistin, two common “last-resort” antibiotics. Tigecycline, a glycylcycline antibiotic with broad-spectrum activity against almost all Gram-positive and Gram-negative bacteria (1), becomes a highly concerned “last-resort” antibiotic for its high frequency of application to treat multidrug-resistant infections. In recent years, resistance to tigecycline has been frequently described and investigated among multidrug-resistant *Enterobacteriaceae* and *Acinetobacter baumannii* strains (2 – 4). Particularly, two publications reported plasmid-hosted mobile *tet*(X3) and *tet*(X4) genes that confer resistance to this antibiotic in 2019, which received worldwide attentions (5, 6). It is worrying that the emergence and dissemination of plasmid-mediated high-level tigecycline resistance genes will greatly decrease the efficacy of this “last-resort” antibiotic.

In addition to *tet*(X) conferring high-level resistance to tigecycline, frequently reported mechanisms associated with resistance to tigecycline include overexpression of AcrAB-TolC, MexXY-OprM, and OqxAB efflux pumps that belong to the resistance-nodulation-division (RND) family (7 – 9). Among them, AcrAB-TolC, as the most common multidrug efflux pump of this category, is composed of three mini-assemblies, the trimeric outer-membrane channel TolC assembly, the trimeric secondary transporter AcrB assembly, and the hexameric periplasmic AcrA assembly linking AcrB and TolC (10). In recent years, the *in situ* structure of full-length *Escherichia coli* AcrAB-TolC was determined by electron cryotomography (11 – 13). Revealed by the Cryo-EM structure of AcrAB-TolC, the tripartite complex structure can effectively ensure that the antibiotic molecules were pumped out of cell instead of remaining in periplasm and simultaneously avoid their re-entrance into the cell (14, 15). It is reported that high-level expression of AcrA and AcrB corresponds to lower intracellular tigecycline concentrations in tigecycline resistant strains (14). Furthermore, there have been a number of reports suggesting mutations of transcription factors that lead to increased expression of the AcrAB-TolC pump, which further leads to tigecycline non-susceptibility (9, 15, 16).

In previous reports, deletion of AcrAB resulted in strains with significantly increased susceptibility to tigecycline and other antibiotics (9). Many mutations of transcription factors, such as *ramA, ramR, soxR,* and *soxS*, are also involved in resistance to tigecycline in an AcrAB-dependent manner (17, 18). In recent years, many mutations in AcrB were reported to promote resistance to multiple antibiotics, including ampicillin, ciprofloxacin, fluoroquinolones, erythromycin, and tetracyclines (19, 20). It can be explained that AcrB is the substrate-binding domain of the pump system, which is regarded as the drug specificity and energy transduction center for the antibiotic transport process (21). Conversely, there has been few research directly associating mutations of AcrA or TolC with antibiotic resistance. Particularly, no report on the role of mutations on AcrA and AcrB conferring resistance to tigecycline have been published so far.

MDR *Klebsiella pneumoniae*, particularly carbapenem-resistant and tigecycline-resistant *K. pneumoniae,* is a major pathogen causing nosocomial infections and is considered a seriously growing global health threat (16). For a long time, the overproduction of AcrAB-TolC and OqxAB, both of which are non-specific active RND efflux pumps, is the most common tigecycline-resistance mechanism in *K. pneumoniae* (16). The role of mutations in AcrAB-TolC on tigecycline resistance, like in other organisms, has not been identified. In this study, a T188A mutation of AcrA in *Klebsiella pneumoniae* was identified, which leads to the reduction of tigecycline susceptibility. Mechanistic investigations confirmed that this mutation leads to reduced susceptibility to tigecycline by increasing tigecycline efflux. Further *in silico* surveillance showed that this mutation is widespread in sequenced *K. pneumoniae* strains. Further correlation analysis of AcrA sequence types and *K. pneumoniae* sequence types suggests the high prevalence of A_188_ type AcrA in hypervirulent multidrug-resistant *K. pneumoniae* sequence types. The emergence of AcrA mutation in T188A suggested that the periplasmic membrane fusion protein, such as AcrA, may play greater roles in antibiotic efflux than originally expected.

## RESULTS

### Antibiotic susceptibility and whole-genome sequencing of a tigecycline-resistant *Klebsiella pneumoniae* strain

A *Klebsiella pneumoniae* 3–94 strain was originally isolated from the sputum sample of a patient suffering from acute myocardial infarction in the Second Hospital of Shandong University and stored at −80°C as part of the clinical strain stock. During routine antimicrobial resistance screening, it was found that this strain is resistant to tigecycline. More comprehensive antibiotic susceptibility tests following the CLSI guidelines was performed using the K-B disk diffusion method, except for polymyxin E for which only the agar dilution method is available. It was found that *K. pneumoniae* 3–94 is a multidrug-resistant strain that is resistant to all β-lactams and two β-lactam/β-lactamase inhibitor combos, trimethoprim, trimethoprim-sulfamethoxazole, a quinolone ciprofloxacin, tetracycline, and tigecycline (Table 1). Additional agar dilution method confirmed *K. pneumoniae* 3–94 is resistant to tigecycline with an MIC value of 4 µg/mL. Whole-genome sequencing (China National Microbiology Data Center accession: NMDC60064229) and subsequent analysis of antibiotic resistance determinants with the CARD database were performed, leading to the identification of mechanisms of resistance to all antibiotics tested but not tigecycline (Table 1), implying that novel mechanisms of tigecycline non-susceptibility may be present.

**TABLE 1:** Antibiotic susceptibility of *K. pneumoniae* 3–94^*a*^

Antibiotic class	Antibiotic	Inhibition zone diameter (mm)	MIC (μg/mL)	ARGs
β-Lactam	AMP	0 (R)	–	*bla*_SHV-1_ *bla*_LAP-2_ *bla*_CTX-M-14_
	PIP	15 (R)	–	
	CAZ	0 (R)	–	
	CTX	19 (R)	–	
	CFZ	10 (R)	–	
	FEP	12 (R)	–	
	CFP	13 (R)	–	
	FOX	11 (R)	–	
β-Lactam /β-lactamase inhibitor	TZP	15 (R)	–	
	AMC	0 (R)	–	
	CZA	30 (S)	–	
Carbapenem	IPM	24 (S)	–	
	MEM	29 (S)	–	
Phenicol	CHL	21 (S)	–	
Fosfomycin	FOS	16 (S)	–	*fosA6*
Aminoglycoside	KAN	27 (S)	–	
	STR	27 (S)	–	
Diaminopyrimidine	TMP	0 (R)	–	*dfrA1*
Diaminopyrimidine sulfonamide	SXT	0 (R)	–	*sul1*
Quinolone	CIP	15 (R)	–	*oqxA qnrS1*
	GAT	20 (S)	–	
Tetracycline	TET	0 (R)	–	*tet(A) tet(C*)
Glycylcycline	TGC	14 (R)	4 (R)	
Polymyxin	PME	**N**	2 (S)	

^*a*^ AMP, ampicilin; PIP, piperacillin; CAZ, ceftazidime; CTX, cefotaxime; CFZ, cefazolin; FEP, cefepime; CFP, cefoperazone; FOX, cefoxitin; TZP, piperacillintazobactam; AMC, amoxicillin-clavulanic acid; CZA, ceftazidime-avibactam; IPM, imipenem; MEM, meropenem; CHL, chloramphenicol; FOS, fosfomycin; KAN, kanamycin; STR, streptomycin; TMP, trimethoprim; SXT, trimethoprimsulfamethoxazole; CIP, ciprofloxacin; GAT, gatifloxacin; TET, tetracycline; TGC, tigecycline; PME, polymyxin E. R, resistant; S, sensitive; –, not tested; N, disk diffusion test not recommended in CLSI standard.

### A point mutation T188A in AcrA results in decreased tigecycline susceptibility

More in-depth analysis of the genomic sequence of *K. pneumoniae* 3–94 revealed that AcrA has a T188A point mutation in comparison with AcrA from the tigecycline-sensitive whole-genome sequenced strain *K. pneumoniae* 2–1 (22) (Genbank accession number: CP031562). This protein is a subunit of the AcrAB-TolC RND-type efflux pump that has been shown to play a major role in tigecycline resistance.

**Fig 1:**
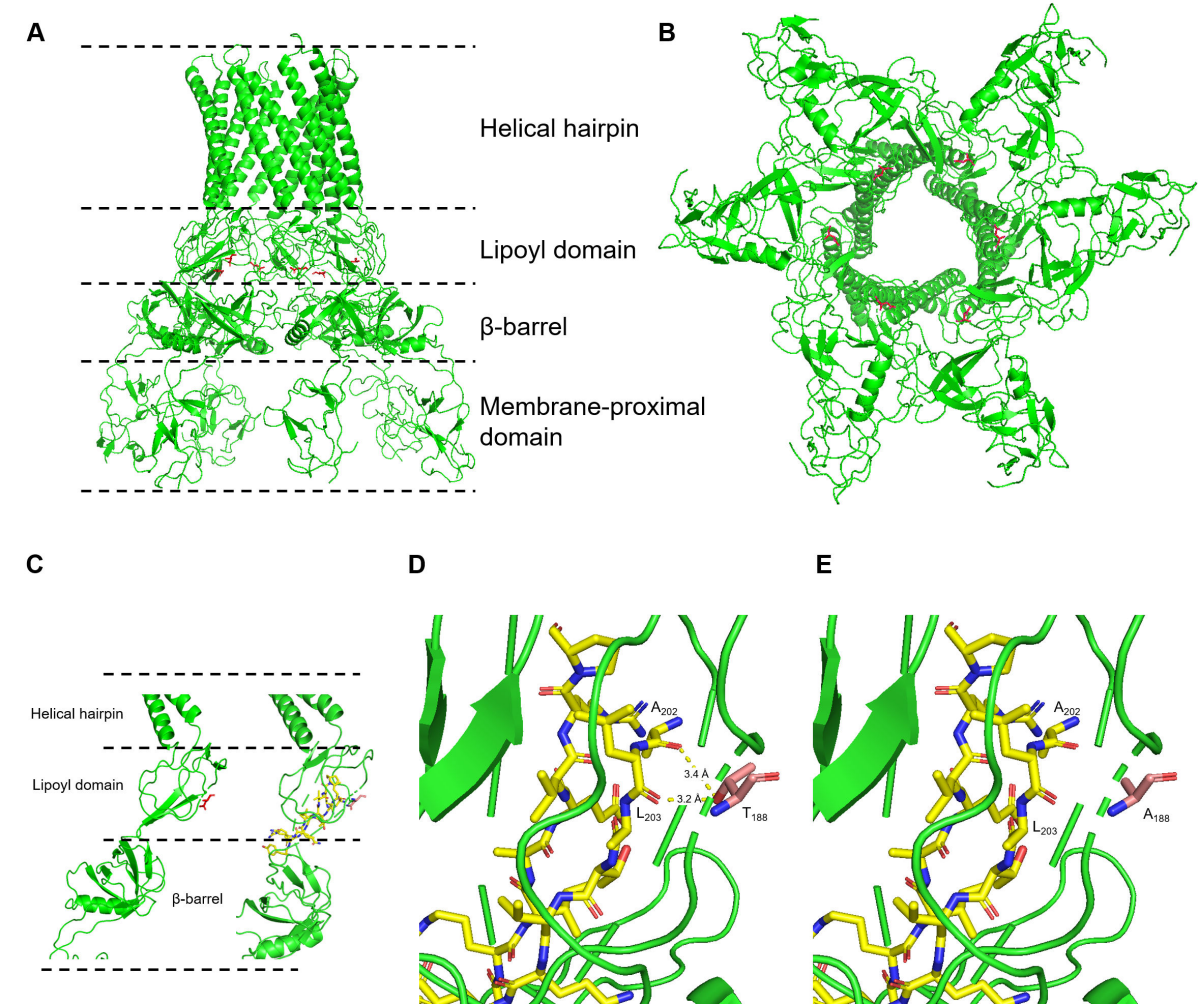
Location of residue 188 on hexameric AcrA assembly. Panel A, side view of *E. coli* AcrA assembly, red sticks indicates residue 188; Panel B, bottom-up view of *E. coli* AcrA assembly, red sticks indicates residue 188; Panel C, residue 188 in relation to the lipoyl β-sheet, red sticks indicate residue 188, β-sheet is represented with cartoon representation on the left side and stick representation on the right side; Panel D, residue T_188_ forms additional hydrogen bonds with the lipoyl β-sheet; Panel E, loss of hydrogen bonds with T188A mutation.

While the structure of *K. pneumoniae* AcrAB-TolC is unavailable, the structure of AcrAB-TolC complex of *Escherichia coli* (PDB ID: 5V5S) was previously solved and published (23). Pairwise sequence alignment with EMBOSS needle showed that all three subunits have good sequence identities between *K. pneumoniae* and *E. coli*: AcrA 85.1% (Genbank accession: *E. coli* NP_414996.1, *K. pneumoniae* WP_267695800.1), AcrB 91.4% (Genbank accession: *E. coli* NP_414995.1, *K. pneumoniae* WP_104443262.1), and TolC 83.8% (Genbank accession: *E. coli* NP_417507.2, *K. pneumoniae* WP_048334428.1). Therefore, *K. pneumoniae* AcrAB-TolC is expected to have a similar structure with *E. coli* AcrAB-TolC. In *E. coli*, N_188_ of AcrA that corresponds to T_188_ in *K. pneumoniae* is located on the lipoyl domain. This periplasmic hexameric AcrA assembly connects the inner membrane-bound trimeric AcrB and the outer membrane-bound trimeric TolC, as seen from the side. The formation of two rings, consisting of lipoyl domains and β-barrel domains, can be observed in the funnel-shaped tunnel that connects AcrB-associated membrane-proximal domains and TolC-associated helical hairpin domains (Fig. 1A). A further bottom-up view (inner membrane to outer membrane) revealed that T_188_ is located on the inner surface of the substrate-pumping channel (Fig. 1B). This residue is adjacent to the anti-parallel sheet that forms the major part of the lipoyl domain (Fig. 1C). A closer look at this domain suggests that the hydroxyl group of the T_188_ side chain forms two hydrogen bonds (bond length respectively 3.2 Å and 3.4 Å) with L_203_ and A_202_, two residues on the β-sheet (Fig. 1D). Upon mutation of T_188_ to A_188_, these two hydrogen bonds no long exist, increasing the flexibility of the lipoyl domain. Therefore, it is hypothesized that the AcrA T188A mutation may have an impact on its structure and may lead to the increase of tigecycline export which further decreases tigecycline susceptibility.

To verify this hypothesis, the *acrAB* gene cluster was deleted from a laboratory-maintained *K. pneumoniae* S1 strain using CRISPR-Cas9 methods as previously published (24). As expected, the *acrAB* deletion strain is susceptible to tigecycline. Re-introduction of *acrA* and *acrB* from *K. pneumoniae* 3–94 cloned on pACYC184 (*K. pneumoniae* S1 Δ*acrAB* + pACYC184*acrA*_A188_ *acrB*) led to resistance to tigecycline (Table 2). When reverting A_188_ to T_188_ (*K. pneumoniae* S1 Δ*acrAB* + pACYC184*acrA*_T188_ *acrB*, Table 2), the MIC values dropped from 4 to 2.5 μg/mL, suggesting improved susceptibility to tigecycline. This is in consistence with the hypothesis that T188A mutation in AcrA led to decreased tigecycline susceptibility.

**TABLE 2:** Tigecycline susceptibility of constructed strains^*a*^

Strain	MIC (μg/mL)
*E. coli* ATCC25922	0.125 (S)
*K. pneumoniae* 2–1	1 (S)
*K. pneumoniae* S1Δ*acrAB*	0.125 (S)
*K. pneumoniae* S1Δ*acrAB* + pACYC184	0.25 (S)
*K. pneumoniae* S1Δ*acrAB* + pACYC184*acrA*_A188_ *acrB*	4 (R)
*K. pneumoniae* S1Δ*acrAB* + pACYC184*acrA*_T188_ *acrB*	2.5 (R)

^*a*^ S, sensitive; R, resistant.

**Fig 2:**
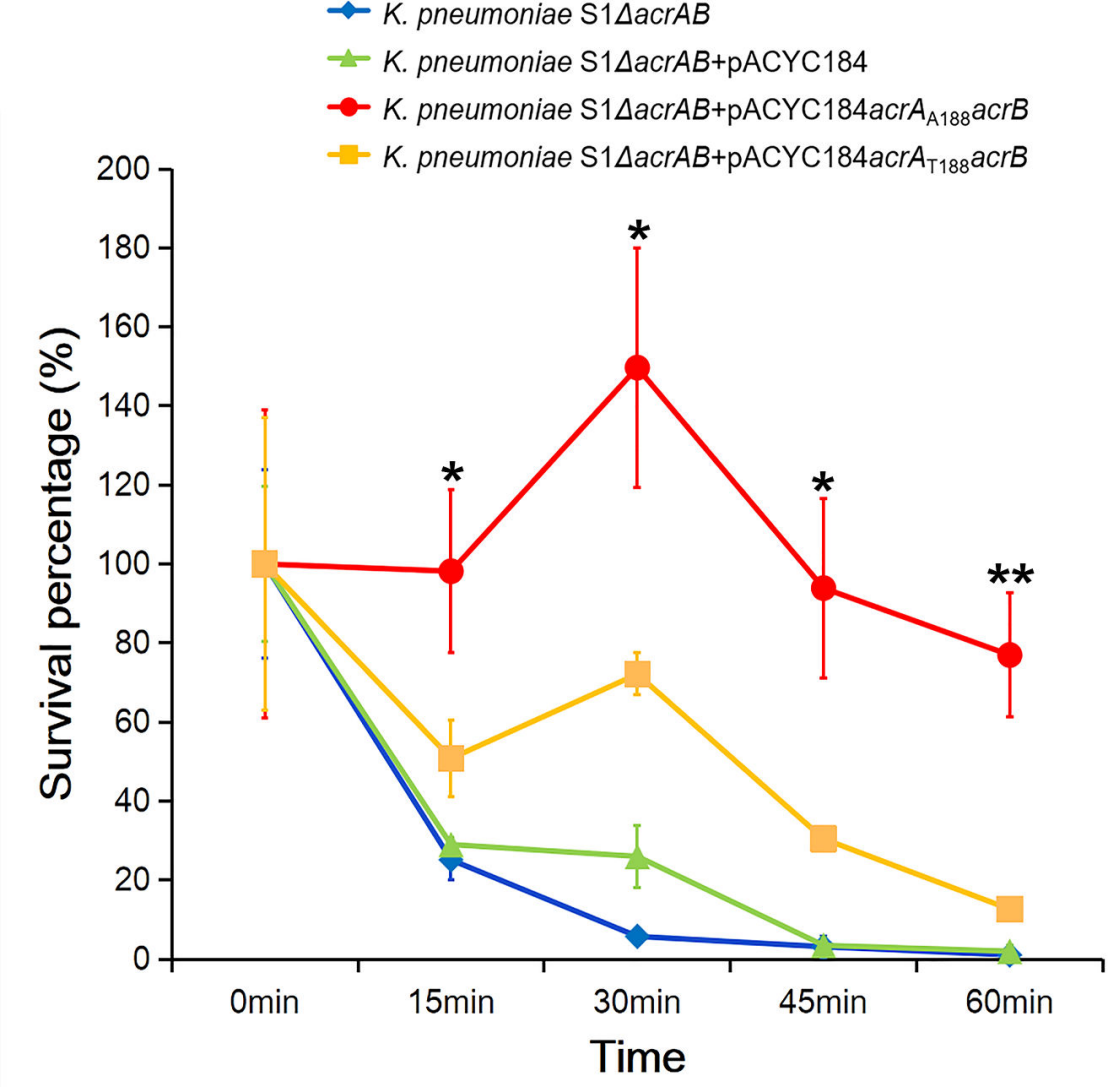
Survival assays of *K. pneumoniae* in the presence of tigecycline. Error bars indicate standard errors. *P < 0.05; **P < 0.01. *P*-values are calculated between *K. pneumoniae* S1Δ*acrAB* + pACYC184*acrA*_A188_ *acrB* and *K. pneumoniae* S1Δ*acrAB* + pACYC184*acrA*_T188_ *acrB* strains.

To further confirm this hypothesis, survival assays were performed, to find out whether T188A mutation in AcrA leads to better tolerance to tigecycline (Fig. 2). When strains were grown in LB medium containing 16 µg/mL of tigecycline, *acrAB* knockout strains showed poor growth. Introducing *acrAB* from *K. pneumoniae* 2–1 (*K. pneumoniae* S1 Δ*acrAB* + pACYC184*acrA*_T188_ *acrB*) showed improved growth, while pACYC184 alone cannot, most likely due to increased *acrAB* levels as a result of multiple plasmid copies. Mutation of T188 to alanine (*K. pneumoniae* S1 Δ*acrAB* + pACYC184*acrA*_A188_ *acrB*) further significantly improved tigecycline tolerance and growth, clearly suggesting that T188A leads to reduced tigecycline susceptibility, and improved tolerance.

### AcrA T188A mutation leads to reduced tigecycline susceptibility by increasing tigecycline efflux

**Fig 3:**
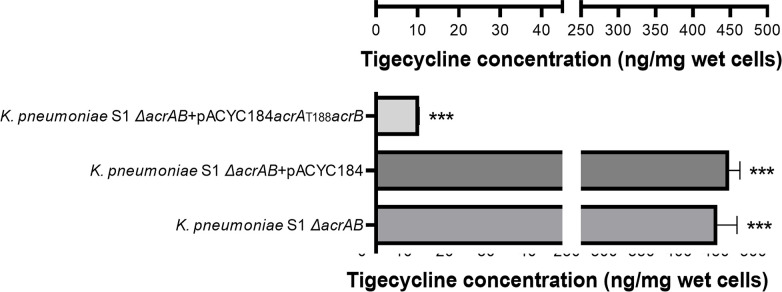
Intracellular tigecycline concentrations of *K. pneumoniae* strains. Error bars indicate standard errors. **P < 0.01; ***P < 0.001. *P*-values are calculated between *K. pneumoniae* S1Δ*acrAB* + pACYC184*acrA*_A188_ *acrB* and other *K. pneumoniae* strains.

In order to find out whether the AcrA T188A mutation reduces tigecycline susceptibility by increasing tigecycline efflux, *K. pneumoniae* strains were incubated in the presence of tigecycline, and intracellular tigecycline levels were measured with high-performance liquid chromatography (HPLC). The *K. pneumoniae* Δ*acrAB* strain had significantly higher intracellular tigecycline levels, suggesting impaired tigecycline efflux by deleting *acrAB* (Fig. 3). Introducing pACYC184 had no impact on intracellular tigecycline levels. Introducing *acrAB* with pACYC184 to *acrAB* knockout strain led to a 45-fold decrease on intracellular tigecycline concentrations. This is in agreement with the findings made with survival assays (Fig. 2). In particular, mutating residue 188 from threonine to alanine led to a 3-fold further reduction of intracellular tigecycline concentration. This is a strong suggestion that AcrA T188A led to significantly increased tigecycline efflux, which further supports the finding that T188A mutation in AcrA led to increase of tigecycline tolerance.

### Widespread presence of T188A mutation in *K. pneumoniae*

**Fig 4:**
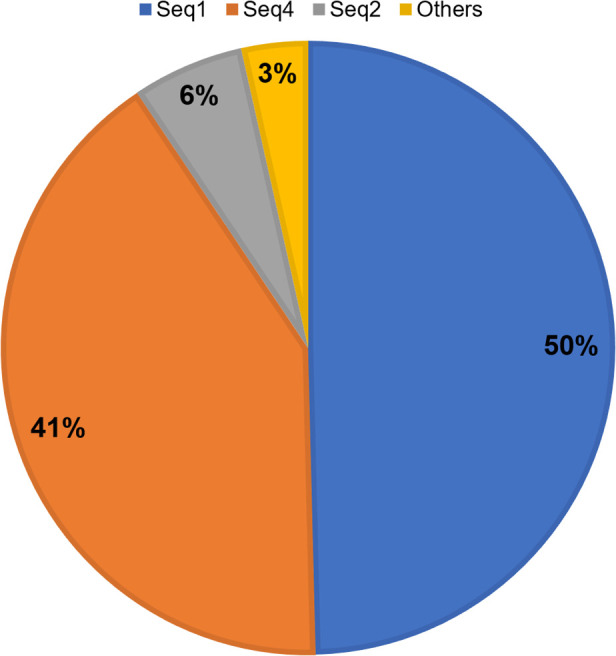
Prevalence of AcrA sequence types in *K. pneumoniae*.

The prevalence of T188A mutation in AcrA was investigated in 14,778 *K. pneumoniae* strains whose genomic sequences are available from Genbank. AcrA-coding genes were found in 14,776 strains. Only 233 different AcrA sequence types were found in all 14,776 *K. pneumoniae* strains, of which 3 top sequence types account for 96.49% of the sequences (Fig. 4). To our surprise, only 6,059 (41.01%) genomes encode AcrA with the same sequence as tigecycline-sensitive *K. pneumoniae* ATCC13883 and *K. pneumoniae* 2–1 (seq4), whereas 7,330 (49.61%) genomes encode AcrA with the same sequence as *K. pneumoniae* 3–94 reported in this work (seq1). Of all genomes, 8,537 (57.78%) encode AcrA with A_188_, whereas 6,191 (41.91%) encode AcrA with T_188_ (Table S1). This finding suggests that the T188A mutation that lead to reduced tigecycline susceptibility is highly prevalent in *K. pneumoniae*, which may imply that the tigecycline effectiveness in treating *K. pneumoniae* infections has already been reduced with the high prevalence of a tigecycline tolerant AcrA sequence type.

The sequence types (STs) of all analyzed *K. pneumoniae* genomes were determined with the MLST algorithm (Table S2), and the distribution of *K. pneumoniae* STs of genomes encoding *K. pneumoniae* ATCC13883 type AcrA (seq4, T_188_) or *K. pneumoniae* 3–94 type AcrA (seq1, A_188_) was analyzed. It is to our surprise finding that *K. pneumoniae* ATCC13883 type AcrA is primarily encoded by only a few STs, whereas *K. pneumoniae* 3–94 type AcrA is encoded by a large variety of STs (Table 3). A more in-depth look found that *K. pneumoniae* strains of clonal group (CG) 258, the most prevalent *K. pneumoniae* type that is characterized by multidrug resistance (25), primarily carry *K. pneumoniae* ATCC13883 type AcrA (99.22% of all CG258 *K. pneumoniae*). Hypervirulent STs, ST23 (98.11%), ST29 (88.98%), ST86 (97.30%), and ST65 (97.37%) mostly encode *K. pneumoniae* 3–94 type AcrA. ST101 (99.52%) and ST307 (99.39%) *K. pneumoniae* that are emerging high risk strains mostly also carry *K. pneumoniae* 3–94 type AcrA (26, 27). Another important group, CG15 *K. pneumoniae* containing ST14 and ST15, carries both *K. pneumoniae* ATCC13883 type and *K. pneumoniae* 3–94 type AcrAs. ST14 mostly carry *K. pneumoniae* 3–94 type AcrA, whereas ST15 mostly carry *K. pneumoniae* ATCC13883 type AcrA. ST20 (97.83%) that is implicated in tigecycline resistance, and ST290 (100.00%) to which *K. pneumoniae* 3–94 belongs encode *K. pneumoniae* 3–94 type AcrA. These findings clearly suggest that AcrA sequence types and *K. pneumoniae* sequence types are closely related and that the high-tigecycline-resistance type AcrA is mostly carried by strains with high virulence, and the low-tigecycline-resistance type AcrA is carried by prevalent strains with high antibiotics resistance.

**TABLE 3:** Sequence types (STs) of *K. pneumoniae* with *K. pneumoniae* ATCC13883 type AcrA (T_188_) and *K. pneumoniae* 3–94 type AcrA (A_188_)^*a*^

Strains	ST	Count	Note
*K. pneumoniae* ATCC13883 type	ST11	2,058	CG258
	ST258	1,369	CG258
	ST15	823	CG15
	ST512	577	CG258
	ST395	141	
	ST37	135	
	ST437	128	CG258
	ST340	121	CG258
	Others	706	
*K. pneumoniae* 3–94 type	ST307	492	
	ST101	418	
	ST16	355	
	ST23	311	Hypervirulent
	ST14	297	CG15
	ST231	233	
	ST45	229	
	ST17	188	
	ST37	130	
	ST35	127	
	ST29	113	Hypervirulent
	ST86	108	Hypervirulent
	ST405	104	
	ST20	92	
	ST48	87	
	ST152	82	
	ST39	75	
	ST65	75	Hypervirulent
	Others	3,498	

^*a*^ Only STs accounting for over 1% of all genomes were listed.

## DISCUSSION

Tigecycline is considered a “last-resort” antibiotic that is widely used when efficacies of other antibiotics are severely impaired due to antibiotic resistance. The putative devastating consequences of resistance to this antibiotic made its resistance a hot topic for research and discussion. Among these researches, mutations in transcription factors governing the expression of *acrAB*, such as AcrR and RamR (28), can lead to improved expression of AcrAB-TolC efflux pump, which, in turn, results in tigecycline resistance. However, although reports on AcrAB-TolC mutation that led to antibiotic resistance have been available (21), possible role of AcrAB-TolC sequence polymorphism in tigecycline has not been recognized.

This work provides evidence that mutation of T_188_ to A_188_ in AcrA led to decreased tigecycline susceptibility by increasing tigecycline efflux. This conclusion is supported by analysis of MICs, survival assays under tigecycline stress, and measurements of intracellular tigecycline concentrations following tigecycline treatment. These consistent experimental observations ensure the validity of the discovery made in this work, which appears to be a new mechanism for tigecycline non-susceptibility. By inspection of the structure of AcrAB-TolC homolog in *E. coli*, we found that this mutation leads to the removal of two hydrogen bonds formed between the hydroxyl group of threonine and a β-sheet that apparently plays a major role in structural rigidity of the lipoyl domain. The removal of hydrogen bonds is predicted to increase the flexibility of the lipoyl domain that is part of the funnel-shaped efflux tunnel of the AcrAB-TolC complex. Weeks et al. also reported that they observed compensatory alterations in the AcrA β-barrel and lipoyl domains when AcrB β-hairpin mutant appears defective in TolC-docking domain. They then explained that the two central domains of AcrA directly interact with AcrB to stabilize the complex and probably permit TolC aperture to transit from closed to open state for drug expulsion by influencing TolC recruitment (20). It is reasonable that the increase of flexibility of the tunnel may, in turn, improve the accommodation of tigecycline molecules, which may be in a variety of orientations and get “stuck” in a rigid tunnel, particularly when a large number of molecular are exported simultaneously. Therefore, although without further experimental structural confirmation, we suspect that the structural basis for increased tigecycline efflux is the improvement of efflux tunnel flexibility.

*K. pneumoniae* is a clinically significant pathogen that is involved in the development of severe life-threatening diseases including pneumonia, bacteremia, pyogenic liver abscess, etc. (29). Its particularly concerning property—extremely strong antibiotic resistance—made it a member of the ESKAPE group that receives special attention for potentials to elicit strong health damage (30). The wide antibiotic resistance spectrum of *K. pneumoniae* severely limits medical options. It is often a necessity to apply riskier strategies such as relying on “last-resort” antibiotics (31). Therefore, the mechanism found in this work that reduces tigecycline efficacy is more important than it normally is, as it hampers one of the few reliable options to treat *K. pneumoniae* infections.

The finding that the T188A mutation is widespread in publicly available *K. pneumoniae* genomes rings an alarming bell that the genotype leading to tigecycline non-susceptibility is already prevalent. This is echoed by previous surveillance studies: 27.8% of studied Enterobacterales were found to be tigecycline resistant in a recent report (32), and meta-analysis of surveillance studies suggests 5.1% of isolated bloodstream *K. pneumoniae* strains are resistant to tigecycline (33). Although genotypes do not necessarily translate into antibiotic resistant phenotypes, it is still worth of concerns, particularly in the context that tigecycline non-susceptibility genotypes appear expanding or even dominating. The very limited number of sequence types for AcrA also hints that this protein appears to be quite conserved, and genotypes could be quite stable once dominating. Correlation analysis of AcrA sequence type and *K. pneumoniae* sequence type suggests these two sequence types are closely linked, and hypervirulence *K. pneumoniae* types usually carry the high-tigecycline-resistance type AcrA. Although the currently prevalent multidrug-resistant sequence types appear to still carry the low-tigecycline-resistance type AcrA, it is likely that hypervirulence *K. pneumoniae* carrying high-tigecycline-resistance type AcrA will reduce the efficacy of tigecycline and reshapes the *K. pneumoniae* sequence type landscape in clinical settings when tigecycline is used more and more frequently. In recent years, it is reported that classical XDR *K. pneumoniae* strains evolved into XDR hypervirulent *K. pneumoniae* through the acquisition of pLVPK-like virulence plasmids (34). Moreover, *K. pneumoniae* ST307 was reported to be the predominant clone type among tigecycline- and carbapenem-resistant *K. pneumoniae* stains in South Korea (16). The high prevalence of high-tigecycline-resistance AcrA type in hypervirulent *K. pneumoniae* is particularly worrisome, as these pathogens elicit greater health damage, and reducing efficacy of one of the last medical options could be devastating.

AcrB, rather than AcrA, has commonly been considered a key subunit deciding efflux efficiencies, as it is the substrate-binding subunit that transports substrates, many antibiotics included, across the inner membrane (21). Instead, AcrA is often viewed as a “connector” that shuttles substrates across the periplasm. Considering the diameter of the tunnel in AcrA is generally bigger (~4–5 nm) than the sizes of antibiotics, it appears reasonable to rule AcrA out as a rate-limiting step for substrate efflux. This work suggests otherwise. Although it may be true that AcrB plays a more important role in substrate recognition, potential impacts of AcrA sequence polymorphism should not be neglected. This may also apply to TolC that governs substrate efflux across the outer membrane.

In conclusion, a T188A mutation of the AcrA subunit of AcrAB-TolC complex was found in *K. pneumoniae*. Evidences support that this mutation leads to reduced tigecycline susceptibility by elevating tigecycline efflux. Further surveillance confirms that this mutation is widespread in sequenced *K. pneumoniae* genomes. To the best of our knowledge, no report of AcrA mutation on tigecycline resistance in *K. pneumoniae* has been previously reported. It is suspected that mutations of the AcrAB-TolC complex may play a larger role in determining antibiotic susceptibility than previously expected.

## MATERIALS AND METHODS

### Strains used in this study

*K. pneumoniae* 3–94 is a strain in the clinical strain stock of Second Hospital of Shandong University. *K. pneumoniae* S1 and *K. pneumoniae* 2–1 are laboratory maintained strains. Sequence data of the two strains were deposited in Genbank, with accession numbers of CP103062 and CP031562.

### Antibiotic susceptibility tests

Antibiotic susceptibility tests were performed according to CLSI or EUCAST guidelines (35, 36). The antibiotics tested by the Kirby-Bauer disk diffusion assays included ampicillin, piperacillin, ceftazidime, cefotaxime, cefazolin, cefepime, cefoperazone, cefoxitin, chloramphenicol, fosfomycin, imipenem, meropenem, kanamycin, streptomycin, trimethoprim, trimethoprim-sulfamethoxazole, ciprofloxacin, gatifloxacin, tetracycline, tigecycline, piperacillin-tazobactam, amoxicillin-clavulanate, and ceftazidime-avibactam. The antibiotics tested by the broth microdilution method included polymyxin B and tigecycline. For tigecycline, a modification of the method was used by setting the antibiotic concentration gradient to (in μg/mL) 64, 32, 16, 8.5, 8, 7.5, 7, 6.5, 6, 5.5, 5, 4.5, 4, 3.5, 3, 2.5, 2, 1.5, 1, 0.5, 0.25, and 0.125 in order to better observe changes in drug susceptibility. The quality control strain is *E. coli* ATCC25922.

### Whole-genome sequencing, assembly, and annotation

Bacterial genomic DNA was extracted using the bacterial genome DNA rapid extraction kit (Mei5 Biotechnology Co., Ltd., Beijing, China) and sequenced at Novogene Technology Co. Ltd, Beijing, China, with an Illumina NovaSeq 6000 platform (Illumina, Inc., San Diego, CA, USA) at PE150 mode and 200 × coverage. SPAdes version 3.15.4 was used for genome assembly (37, 38). QUAST version 4.6.0 was used to evaluate the quality of genome assembly (39). RGI version 6.0.1 with CARD version 3.2.6 was used to annotate the resistance genes (40). Assembled genomic sequences are deposited in China National Microbiology Data Center (NMDC) with accession numbers NMDC60064229.

### Genetics

Construction of *acrAB* knockout *K. pneumoniae* strain was performed using the CRISPR-Cas9 system as previously reported (24). The engineering plasmids used in the knockout process are shown in Table S3. The spacer and single-stranded DNA repair template sequences designed for gene knockout are shown in Table S4. The sequence of the primers required for plasmid construction and the knockout validation are shown in Table S5.

To express *acrAB* from *K. pneumoniae* 2–1 and *K. pneumoniae* 3–84 in *K. pneumoniae* S1 Δ*acrAB*, genes were amplified and cloned into pACYC184 whose selection marker was replaced with apramycin-resistant *aac (2)IV*. Cloning was performed with MultiF Seamless Assembly Mix (ABClonal Inc., China). Ligated plasmids were transformed into *E. coli* DH5α and selected on LB plates containing 30 mg/L apramycin. Correct constructs were transformed into *K. pneumoniae* S1 Δ*acrAB* with electroporation. Primers used for expressing *acrAB* can be found in Table S5.

### Survival assay

*K. pneumoniae* S1 Δ*acrAB*, *K. pneumoniae* S1 Δ*acrAB* + pACYC184, *K. pneumoniae* S1 Δ*acrAB* + pACYC184*acrA*_A188_ *acrB*, and *K. pneumoniae* S1 Δ*acrAB* + pACYC184*acrA*_T188_ *acrB* strains were assayed for survivability under TGC stress similar to previous published literature (41). Specifically, cells were grown to exponential phase (OD_600_ = 0.5) in LB liquid medium. Four microliters of the culture was diluted by 10^2^- to 10^6^-fold and grew on LB plates for CFU calculation. Tigecycline at a final concentration of 16 µg/mL was added to liquid culture to challenge cells. Culture was removed every 15 min for CFU calculation as mentioned above. The survival rate is calculated by dividing the CFU number post-challenge and pre-challenge. Three biological replicates were performed.

### Determination of intracellular TGC content

Determination of intracellular tigecycline levels was performed by the HPLC method similar to previously reported protocol (14). *K. pneumoniae* S1 Δ*acrAB*, *K. pneumoniae* S1 Δ*acrAB* + pACYC184, *K. pneumoniae* S1 Δ*acrAB* + pACYC184*acrA*_A188_ *acrB*, and *K. pneumoniae* S1 Δ*acrAB* + pACYC184*acrA*_T188_ *acrB* strains were grown in LB liquid medium to exponential phase (OD_600_ = 0.5) and centrifuged at 2000 × *g* for 5 min. Fifty milligrams of cell pellets was suspended in 5 mL of 0.05 M PBS (pH 7.0) and incubated at 37°C for 10 min. Sterile TGC was added to cell suspensions at a final concentration 50 µg/mL and incubated at 37°C for 5 min. Two hundred and fifty microliters of DMSO was subsequently added, followed by further incubation at 37°C for 5 min. Cells were further pelleted by centrifugation at 2,000 × *g* for 5 min and washed in 5 mL of pre-chilled 0.05 M PBS (pH 7.0) for 3 times. One milliliter of 0.1 M glycine-hydrochloric acid (pH 3.0) was used to suspend cells, followed by incubation for 16 h. Cells were removed by centrifugation at 10,000 × *g* for 10 min. The supernatant was further sterilized by passing through 0.22 µm filters. Tigecycline content was assayed with HPLC equipped with a Sepax GP-C18 column. Diammonium hydrogen phosphate-triethylamine-methanol salt solution (50:1:49) was used as the mobile phase at a flow rate of 1.0 mL/min. Column temperature of 30°C and detection wavelength of 248 nm were used. Three replicates were performed.

### Analysis of *acrA* sequence types and sequence types

Genomic sequences of 14,778 *K. pneumoniae* strains were downloaded from Genbank. Genemark S-2 version 1.14_1.24_lic was used to predict protein-coding genes from genomes (42). AcrA homologs were identified using blastp version 2.13.0 + by searching for similar proteins with AcrA from *K. pneumoniae* 2–1 (43). AcrA homologs were further aligned by Clustal X version2.1 (44), and sequence types were analyzed. Sequence types of *K. pneumoniae* were analyzed with MLST version 2.19.0 authored by Seemann T (https://github.com/tseemann/mlst) using PubMLST database (45).

### Statistics

Comparisons of survival rates and intracellular tigecycline levels were made using two-tailed Student’s *t*-test. *P* < 0.05 was considered statistically significant.

## SUPPLEMENTAL MATERIAL

Tables S3 to S5 spectrum.02030-23-s0001.docx Plasmids and primers for construction.

Table S1 spectrum.02030-23-s0002.xlsx Number and sequences of AcrA sequence types in *K. pneumoniae*.

Table S2 spectrum.02030-23-s0003.xlsx Sequence types of *K. pneumoniae* and AcrA sequence types.

ASM does not own the copyrights to Supplemental Material that may be linked to, or accessed through, an article. The authors have granted ASM a non-exclusive, world-wide license to publish the Supplemental Material files. Please contact the corresponding author directly for reuse.
