# Supplementary material for: A widespread single amino acid mutation in AcrA reduces tigecycline susceptibility in Klebsiella pneumoniae
Source: Microbiol Spectr. 2023 Nov 30;12(1):e02030-23. doi: 10.1128/spectrum.02030-23 (PMC10782974; doi:10.1128/spectrum.02030-23)
Supplement: Tables S3 to S5 — Plasmids and primers for construction. [file spectrum.02030-23-s0001.docx]

**Table S3. Plasmids and strain used in this work.**

| **Plasmids and strain** | **Description** | **Source or reference** |
| --- | --- | --- |
| pSGKP-*spe* | Plasmid expressing sgRNA, spectinomycin resistance | (24) |
| pCasKP-*hph* | Plasmid encoding Cas9 protein, λ Red recombination proteins, hygromycin B resistance | (24) |
| pSET152 | Streptomyces integrative vector, the resistance gene on the plasmid is used for plasmid modification, apramycin resistance | This study |
| pSGKP-*apr* | Derivative of pSGKP-*spe*, apramycin resistance | This study |
| pACYC184 | pACYC184 plasmid, selection marker replaced by *apr*. | This study |
| *Escherichia coli* DH5α | Model strain for plasmid construction | This study |

**Table S4. Spacer and repair template sequences for gene knockout by CRISPR.**

| **Name** | **Sequence (5’-3’)** | **Reference** |
| --- | --- | --- |
| *acrB*-spacer | CGATATTTCGGACTACGTGG | This study |
| *acrA*-spacer | CTGAAGCAGGAGCTAGCCGA | This study |
| *acrB*-ssDNA | CATCAGAACAAACCAAGTCTTAACTTAAACAGGAGCCGTTAAGAC  TGGTGGAAGCGATCATCCTGGTGTTCCTGGTCATGTATCTGTTCC | This study |
| *acrA*-ssDNA | CGCCGTTGCGACGGCGCAGCAGAGCAATGCGGCCGTGGTCGCGGCGTGTTACCCGTACGCCGCGCGGCGACGCCAGCGTCATGGTAGTGG | This study |

**Table S5. Primers used for plasmid construction.** Underlined parts indicate gene homology arms.

| **Primer name** | **Sequence (5’-3’)** | **Application** | **Reference** |
| --- | --- | --- | --- |
| pSGKP-F | TTTCGCCATTCGTATTGCACACTCTTCCTTTTTC | pSGKP-*apr* construction | This study |
| pSGKP-R | CTCGCCAGTCGATTGGCTGACTGTCAGACCAAGTTTACTC |  |  |
| *apr*SG-F | GAGTAAACTTGGTCTGACAGTCAGCCAATCGACTGGCGAG |  | This study |
| *apr*SG-R | AATATTGAAAAAGGAAGAGTGTGCAATACGAATGGCGA |  |  |
| pSG*acrB*-spacer F | TAGTCGATATTTCGGACTACGTGGGTTTTAGAGCTAGAAATAGC | Construction of pSGKP-*apr* plasmid containing the spacer of *acrB* | This study |
| pSG*acrB*-spacer R | CCACGTAGTCCGAAATATCGACTAGTATTATACCTAGGACTGAGC |  |  |
| K-*acrB* F | CCATCAGAACAAACCAAGTC | Verification the deletion of *acrB* | This study |
| K-*acrB* R | TTCAGCATCGTGGCGCACA |  |  |
| pSG*acrA*-spacer F | TAGTCTGAAGCAGGAGCTAGCCGAGTTTTAGAGCTAGAAATAGC | Construction of pSGKP-*apr* plasmid containing the spacer of *acrA* | This study |
| pSG*acrA*-spacer R | TCGGCTAGCTCCTGCTTCAGACTAGTATTATACCTAGGACTGAGC |  |  |
| K-*acrA* F | AACAGAGGGTTAACGCCT | Verification the deletion of *acrA* | This study |
| K-*acrA* R | CGTCAGTGACCAGCCATT |  |  |
| Spacer test | TTGCAGACTACGGGCCTAA | Verification the spacer sequence inserted in pSGKP-*apr* plasmid | This study |
| M13 R | CAGGAAACAGCTATGACC |  |  |
| pCaSKP-LF | GCGTGAGCCATGAGAACGAA | Verification the plasmid loss | This study |
| pCaSKP-LR | CCAGTGGACAAACTATGCC |  |  |
| pSGKP-LF | CGTTGATTGTTTGTCTGCG | Verification the plasmid loss | This study |
| pSGKP-LR | CTACCAAGGCGAAGAATCTT |  |  |
| pACYC184 F | TCAGGTTGAGCATCATTAACCCTCCCACATAACCAG | Construction of pACYC184*acrA*_T188_*acrB* plasmid | This study |
| pACYC184 R | GCAACTGATTCTGGATGTTGCTAACCATGGATCCATGG |  |  |
| *acrA_T188A_* F | CCATGGATCCATGGTTAGCAACATCCAGAATCAGTTGC |  | This study |
| *acrA_T188A_* R | TCGATAAAGAAATTAGGCATTTAAGACTTGGTTTGTTC |  |  |
| *acrB* F | GAACAAACCAAGTCTTAAATGCCTAATTTCTTTATCGA |  | This study |
| *acrB* R | CTGGTTATGTGTGGGAGGGTTAATGATGCTCAACCTGA |  |  |
| Test F1 | GCTGTGAGCGCTTTGCCTTG |  | This study |
| Test R1 | GGTTCAAAGAGTTGGTAGCT |  |  |
| *acrA* F | GCGGCCGGATTGGTAAATCCACCGTGACCG | Construction of pACYC184*acrAacrB* plasmid | This study |
| *acrA* R | CGGTCACGGTGGATTTACCAATCCGGCCGC |  |  |
| Test F2 | AGGCCGGCGTCTCCCTGTAT |  | This study |
| Test R2 | ACAGGGTGTGATCCGGGTTC |  |  |
